# C(P)XCG Proteins of *Haloferax volcanii* with Predicted Zinc Finger Domains: The Majority Bind Zinc, but Several Do Not

**DOI:** 10.3390/ijms25137166

**Published:** 2024-06-28

**Authors:** Deniz Üresin, Jonathan Schulte, Nina Morgner, Jörg Soppa

**Affiliations:** 1 Institute for Molecular Biosciences, Goethe University, 60438 Frankfurt, Germany; ueresin@bio.uni-frankfurt.de; 2 Institute of Physical and Theoretical Chemistry, Goethe University, 60438 Frankfurt, Germany; schulte@chemie.uni-frankfurt.de (J.S.); morgner@chemie.uni-frankfurt.de (N.M.)

**Keywords:** archaea, *Haloferax volcanii*, zinc finger, metal-binding proteins, small proteins, microproteins, ESI, mass spectrometry, C(P)XCG motif, AlphaFold2

## Abstract

In recent years, interest in very small proteins (µ-proteins) has increased significantly, and they were found to fulfill important functions in all prokaryotic and eukaryotic species. The halophilic archaeon *Haloferax volcanii* encodes about 400 µ-proteins of less than 70 amino acids, 49 of which contain at least two C(P)XCG motifs and are, thus, predicted zinc finger proteins. The determination of the NMR solution structure of HVO_2753 revealed that only one of two predicted zinc fingers actually bound zinc, while a second one was metal-free. Therefore, the aim of the current study was the homologous production of additional C(P)XCG proteins and the quantification of their zinc content. Attempts to produce 31 proteins failed, underscoring the particular difficulties of working with µ-proteins. In total, 14 proteins could be produced and purified, and the zinc content was determined. Only nine proteins complexed zinc, while five proteins were zinc-free. Three of the latter could be analyzed using ESI-MS and were found to contain another metal, most likely cobalt or nickel. Therefore, at least in haloarchaea, the variability of predicted C(P)XCG zinc finger motifs is higher than anticipated, and they can be metal-free, bind zinc, or bind another metal. Notably, AlphaFold2 cannot correctly predict whether or not the four cysteines have the tetrahedral configuration that is a prerequisite for metal binding.

## 1. Introduction

Very small proteins have been neglected for a long time because commonly, a lower limit of 100 codons was used for the annotation of genes to avoid the annotation of many false positives. However, in recent years, it has become evident that very small proteins are ubiquitously distributed in archaea, bacteria, and eukaryotes [1]. Important experimental approaches for the identification of very small proteins were the establishment of ribosomal profiling and the adaptation of the workflow for protein extraction and analysis via mass spectrometry (“peptidomics”) [2,3,4,5]. The field is so new that a common nomenclature does not exist, and very small proteins are called “peptides”, “small peptides”, “µ-peptides”, “µ-proteins”, “sORF-encoded proteins”, or “sORF-encoded polypeptides” (SEPs). In addition, no common definition of the upper size limit exists and often, 50 amino acids (aa), 70 aa, or 100 aa are used. In this contribution, the term µ-proteins and an upper limit of 70 aa will be used. The small size is not a predictor of a common function, in fact, µ-proteins are involved in many different biological processes. In humans, their malfunction has been correlated to many different diseases or developmental disorders. Various reviews are available that summarize different aspects of the features and functions of µ-proteins [6,7,8,9,10,11,12,13,14].

Already 17 years ago, the Oesterhelt group characterized the low molecular weight proteome of the halophilic archaeon *Halobacterium salinarum* [15]. In total, 380 proteins of less than 20 kDa were detected. Most of them had not been included in the genome annotation before and had no annotated function. Remarkably, 20 of these proteins contained two C(P)XCG motifs (C—cysteine, P—proline, G—glycine, X—any amino acid). It was hypothesized that the four cysteines might complex a zinc ion and, thus, that 20 novel small zinc finger proteins with unknown functions were detected. The P is set in parentheses because only one of the two motifs contains a proline at the second position. Many proteins contain slight variations of the standard motifs, e.g., only one of the two motifs has a G at the fifth position, or the distance between the two cysteines is three instead of two amino acids in one of the two motifs, or both motifs are lacking the proline at the second position. Despite these slight variations, these “C(P)XCG-like” proteins will be included in this new family of predicted zinc finger µ-proteins.

One of these *H. salinarum* proteins turned out to regulate (directly or indirectly) the transcription of the *bop* (bacterioopsin) gene and the *crtB1* gene encoding an enzyme involved in carotenoid biosynthesis [16], while the functions of the remaining proteins remained enigmatic.

Zinc finger proteins were first discovered in eukaryotes and were long thought to be confined to this domain. However, about 25 years ago, the first bacterial zinc finger protein was described, i.e., the Ros protein of *Agrobacterium tumefaciens* [17,18]. Ros homologues were later found to be present in many bacterial species, and several representatives thereof have been characterized [19,20]. Although today various bacterial zinc finger proteins are known, their fractions of the bacterial proteomes are very small, and the number of eukaryotic zinc finger proteins is much higher.

The zinc ion in zinc fingers can be complexed by four cysteines (C4 fingers), by three cysteines and one histidine (C3H fingers), or by two cysteines and two histidines (C2H2 fingers). The C2H2 fingers are called “classical” zinc finger motifs because they are more common than the other two types and have been studied intensively. Zinc fingers have been categorized into eight different structural groups [21]. The C2H2 zinc finger domains are typical DNA-binding domains of eukaryotic transcription factors (TFs), and about 700 of these TFs have been found in mammals [22]. Each zinc finger recognizes only a short DNA motif of 3–4 bp; therefore, TFs contain several to many zinc finger domains to enable specific DNA recognition. A “recognition code” for zinc fingers has been established, which enables the generation of artificial TFs for any desired target site [23,24].

Zinc finger domains do not only interact with DNA. In stark contrast, they are very versatile interaction domains that can interact with RNA, other proteins, lipids, and more [21,25,26,27,28]. Therefore, the presence of zinc fingers in a protein does not allow the prediction that it is a DNA-binding TF, but the function of every new zinc finger protein has to be determined experimentally.

The genome of the haloarchaeal model species *Haloferax volcanii* contains 49 genes for putative zinc finger µ-proteins with two C(P)XCG motifs (one protein contains four motifs). Our project, which concentrates on the characterization of these proteins, is part of the Priority Program “Small Proteins in Prokaryotes: an unexplored World” (www.spp2002.uni-kiel.de (accessed on 1 May 2024)). This initiative combines more than 20 groups that concentrate on various aspects of µ-proteins from many different species of archaea and bacteria.

To characterize the C(P)XCG µ-proteins of *H. volcanii*, more than 30 single gene deletion mutants were constructed and their phenotypes were compared to that of the wildtype ([29], unpublished results). The majority of the mutants exhibited a phenotype under at least one condition, e.g., growth on a specific carbon source, swarming, or biofilm formation. The NMR solution structure of the protein HVO_0758 was solved [30]. The structure as well as a biochemical fluorescence assay verified that one zinc ion is complexed by HVO_0758, as predicted based on the presence of two C(P)XCG motifs. In addition, the solution structure of the protein HVO_2753 was also determined [31]. This is the only protein that contains four C(P)XCG motifs and was, therefore, expected to contain two zinc fingers. Unexpectedly, the structure, a zinc titration NMR experiment, and the fluorescence assay revealed that HVO_2753 binds only one zinc ion.

The fact that only two of the three analyzed zinc fingers really bind zinc prompted us to start the current project. The aim was to obtain a comprehensive overview of the zinc-binding ability of the predicted *H. volcanii* zinc finger µ-proteins. This included the homologous production, purification, and quantification of their zinc content. The results should enable an estimation of the predictive power of the bioinformatics prediction that C(P)XCG proteins always complex zinc and contain zinc finger domains. It seemed possible that HVO_2753 is not the only protein with a metal-free “zinc finger” because a few proteins of the bacterial Ros family were also shown to be metal-free. In addition, the bacterial rubredoxin does not bind zinc, but iron. Therefore, for each protein, three possible outcomes were expected: it binds zinc, it binds another metal, or it is metal-free. Here, we report that in total, 14 proteins could be produced, and their zinc content could be determined. In addition, nanoElectrospray Ionisation mass spectrometry (ESI-MS) was used to elucidate the native protein mass to differentiate between metal-free and metal-containing predicted zinc finger proteins.

## 2. Results

### 2.1. Length Distribution of Proteins with C(P)XCG Motifs in Haloferax volcanii

The genome sequence of *H. volcanii* is regularly updated by Friedhelm Pfeiffer (MPI for Biochemistry, Martinsried, Germany). The latest version was searched for the number of annotated proteins with C(P)XCG motifs (www.halolex.mpg.de (accessed on 13 May 2024)). The length distributions of C(P)XCG proteins and of all proteins are summarized in Table 1. No C(P)XCG proteins had a length of less than 30 aa. While eukaryotic zinc finger domains typically have lengths around 30 aa, probably for stand-alone proteins, this length is too small to accommodate an N-terminal region, the first C(P)XCG motif, the linker, the second C(P)XCG motif, and the C-terminal region. By far the highest fractions of C(P)XCG proteins were found in the length classes of 40–50 aa and 50–60 aa, where they make up 17.9% and 18.5% of all proteins, respectively. In stark contrast, CXPXG proteins are extremely scarce in the length class of more than 100 aa (only 0.6%). Therefore, haloarchaeal zinc fingers are mostly found in µ-proteins and, thus, these represent single-domain zinc finger proteins. This is in contrast to eukaryotic zinc finger proteins, which are typically rather large and contain several to many zinc finger domains (mostly classical C2H2 zinc fingers), in addition to various other functional protein domains. Notably, the *H. volcanii* C(P)XCG µ-proteins of up to 70 aa characterized in the present study are not exceptional but are typical for haloarchaea and constitute by far the largest fraction.

### 2.2. Homologous Production and Purification of Predicted Zinc Finger µ-Proteins

At the start of this project, the genome annotation of *H. volcanii* contained 45 genes encoding proteins of up to 70 aa with C(P)XCG motifs (until now, 4 more such genes have been identified; thus, the current number is 49). The two genes *hvo*_0758 and *hvo_2753* had already been characterized previously. The remaining 43 genes were amplified using PCR and cloned into the expression vector pSD1/R1-6 [32], which contains a strong synthetic constitutive promoter. For all genes, two versions were cloned, which encoded either an N-terminal or a C-terminal His_6_-tag, respectively, so that a total of 86 expression plasmids were generated. All plasmids were introduced into *H. volcanii*. If available, the cognate deletion strain was used as a production strain; if the deletion strain was not yet available or the gene was essential, the wildtype H26 was used. For complementation studies, the usage of the respective mutant is mandatory, while for the aim of the current study, an additional non-tagged version of the respective protein in the cell does not compromise the results.

It turned out that only 14 of the 45 proteins (including HVO_0758 and HVO_2753) could be produced in amounts that allowed biochemical characterization. Only very few could be produced with the N-terminal as well as the C-terminal His_6_-tag, underscoring the necessity to attempt both versions. The 45 proteins, their sizes, C(P)XCG motifs, and production levels are summarized in Supplementary Table S1. A total of 35 of the proteins had a proline in one of the two motifs while in 1 protein, both motifs included a proline, and in 10 proteins, both motifs lacked a proline. The glycine at the fifth position was even more variable—9 proteins had the “classical” version with a glycine in both motifs, for 30 proteins, only one motif contained a glycine, and for 7 proteins, both motifs lacked a glycine. We attempted to correlate the successful production in the 14 proteins (or—even more—the production level) with several possible features, including protein size, isoelectric point, or the presence of prolines or glycines in the C(P)XCG motifs. Unfortunately, these attempts failed, so the success of the production of 14 examples and the failure of the production of 31 examples could not be rationalized.

**Figure 1:**
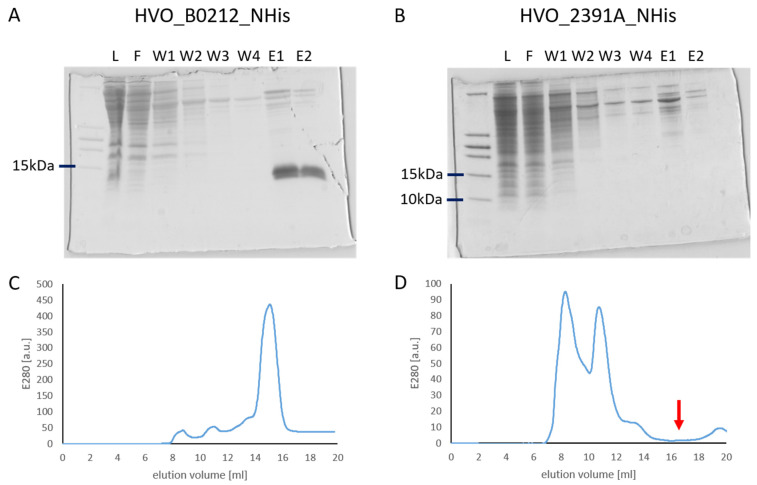
(**A**) Affinity purification of protein HVO_B0212 using nickel chelating sepharose. Aliquots of the following fractions were analyzed on a tricine SDS-PAGE: L—cell lysate, F—flow through, W—wash fractions, and E—elution fractions. (**B**) Tricine SDS-PAGE of the different fractions of an attempt to affinity purify protein HVO_2391A. (**C**) Chromatogram of a size exclusion chromatography of the elution fractions of the affinity isolation shown in (**A**). (**D**) Chromatogram of a size exclusion chromatography of the elution fractions of the affinity isolation shown in (**B**). The red arrow indicates the elution volume of a protein of the size of HVO_2391A-NHis.

Figure 1A exemplifies the successful affinity purification of the protein HVO_B0212_NHis, which had a high production level. In contrast, Figure 1B exemplifies the attempted affinity purification of the protein HVO_2391A_NHis, which could not be produced like the majority of the 45 proteins. Notably, the affinity isolation with nickel chelating sepharose does not only purify the tagged proteins but also additional native proteins of *H. volcanii* that contain runs of histidines, i.e., PitA and Cdc48d [33]. These proteins were taken as internal controls that the isolation procedure was working properly, and only the tagged version of the respective µ-protein was missing (compare lane E1 in Figure 1A,B).

Size exclusion chromatography (SEC) was used as a second purification step to isolate pure monomeric C(P)XCG protein and dispose of native histidine-rich *H. volcanii* proteins and proteins that might have been isolated indirectly based on their interaction with the tagged protein. Figure 1C exemplifies the successful isolation of a protein (HVO_B0212_NHis), while Figure 1D exemplifies the lack of a peak at the expected elution position of a µ-protein (see arrow).

In summary, 12 C(P)XCG µ-proteins in addition to the previously characterized HVO_0758 and HVO_2753 could be produced and purified in amounts that were sufficient for biochemical characterization.

### 2.3. Quantification of Zinc Binding Using a Fluorimetric Assay

**Figure 2:**
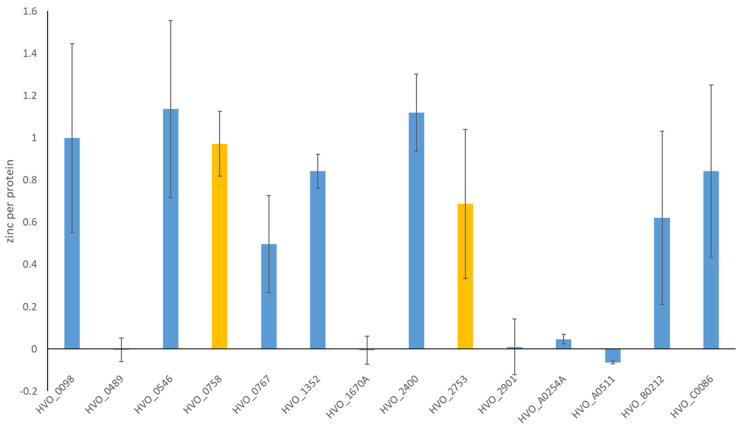
Zinc content of the 14 characterized H. volcanii C(P)XCG µ-proteins. A quantitative assay with the highly zinc-specific fluorophore ZnAF-2F was applied. Mean values of at least three biological replicates and standard deviations are shown. The results of previous studies [30,31] are included and marked in yellow.

Haloarchaea use the so-called Salt-In strategy for osmoadaptation, and their intracellular salt concentration equals that of the high-salt environment. Accordingly, their proteins are adapted to the cytoplasmic high-salt conditions and typically denature under low-salt conditions [34,35,36]. In contrast, the two zinc finger µ-proteins HVO_0758 and HVO_2753 were found to be stable and natively folded at low salt. Therefore, the experimental design to liberate the complexed zinc ion for its quantification did not only include a dialysis against a low-salt buffer but also a proteolytic digestion of the proteins. For the 12 newly isolated proteins of the current study, it was not individually tested whether or not they are stable at low salt. To ensure that the complexed zinc can be quantified, they were isolated under native conditions (see above), then dialyzed against low salt, and hydrolyzed using proteinase K. Then, the zinc content was quantified using the very sensitive and highly zinc-specific fluorophore ZnAF-2F [37]. The results for all 14 proteins are summarized in Figure 2.

For nine proteins between 0.5 and 1.1 equivalent zinc contents were found in at least three biological replicates and, therefore, it was concluded that they are bona fide zinc finger proteins. The values below 1.0 equivalent zinc might either be due to a partial loss of zinc during the two-step isolation procedure of the protein, inaccuracies in the protein quantification method, the variation of the zinc quantification method, or a combination of all three. Remarkably, the zinc content of 5 of the 14 proteins was found to be zero. To avoid the possibility that in these cases, problems with the fluorimetric assay might lead to the failure of zinc detection, one equivalent zinc was added as an internal standard, and in all cases, it could be faithfully detected (Supplementary Figure S1).

Taken together, the majority of 9 of the 14 characterized C(P)XCG proteins indeed bound zinc, but an unexpectedly high fraction of 5 of the 14 predicted zinc finger proteins were found to be zinc-free. Further analyses aimed to differentiate whether they were totally devoid of metals or whether an alternative metal was complexed.

### 2.4. Differentiation of Metal-Free versus Metal-Containing Proteins Using ESI-MS

ESI-MS was applied to determine whether the zinc-free C(P)XCG proteins were totally devoid of metals or whether an alternative metal was bound. Nanoelectrospray Ionization Mass Spectrometry is a very mild method that has been shown in various examples to preserve ion–protein complexes and reliably determine their mass [38,39,40]. Nevertheless, it hasn’t been used to study haloarchaeal proteins previously and, thus, we could not dismiss the possibility that a bound metal might not be retained during the analysis, leading to the erroneous detection of a metal-free mass of the protein. In addition, ESI-MS can only be used in the absence of salt or at very low-salt conditions, and for the newly investigated proteins, it was not known whether or not they retain a possibly bound metal during dialysis against a buffer lacking NaCl. Therefore, the zinc-containing protein HVO_0758 was used as a positive control for method establishment. In addition, for all proteins, two samples were analyzed, one of which was treated with EDTA to remove a putatively bound divalent metal, and only a difference between the two spectra was taken as proof for metal binding.

**Figure 3:**
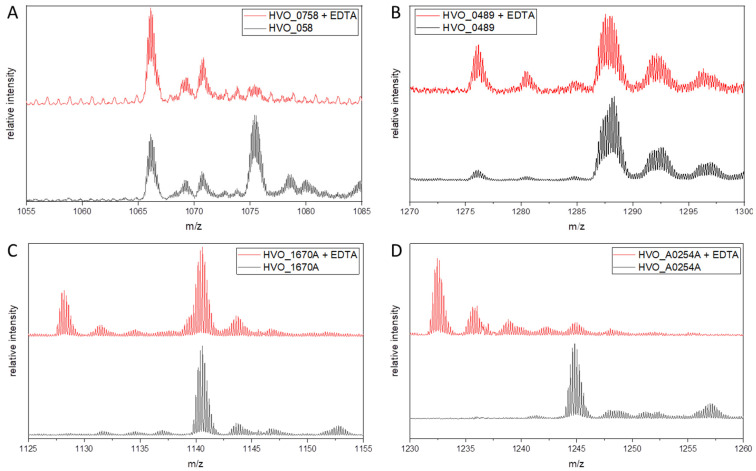
ESI mass spectra of different zinc finger µ-proteins after treatment with EDTA (red) and without treatment (black). (**A**) Analysis of positive control protein HVO_0758. (**B**) Analysis of protein HVO_0489. (**C**) Analysis of protein HVO_1670A. (**D**) Analysis of protein HVO_A0254A.

Figure 3A shows the results for the positive control HVO_0758. Clearly, EDTA treatment resulted in the loss of a peak at an *m*/*z* ratio of around 1075, indicating that this peak represents a zinc-containing holo-protein, while the peak at around 1066 *m*/*z* represents the zinc-free apo-protein. The presence of a 1066 *m*/*z* peak also in the absence of a prior EDTA treatment indicated a partial zinc loss during the analysis. The results for the protein HVO_0489 are shown in Figure 3B. A peak at *m*/*z* 1287 was present regardless of the EDTA treatment; however, a peak at *m*/*z* 1276 was hardly visible in the absence of EDTA but clearly visible after the treatment, indicating the generation of a metal-free apo-protein. The results for HVO_1670A are shown in Figure 3C. The EDTA treatment led to the occurrence of a new peak at 1028 *m*/*z*, indicating that the treatment had led to a partial liberation of a divalent metal ion. The high peak at 1141 *m*/*z* indicated that the metal-containing protein is very stable and neither the EDTA treatment nor the ESI-MS analysis led to a large metal loss. The results were very different for HVO_A0254A (Figure 3D). Without the EDTA treatment, exclusively one peak at 1245 *m*/*z* was found, whereas after EDTA treatment, this peak was totally lost and a new peak at 1232 *m*/*z* arose. Whereas the properties of the three proteins concerning the EDTA treatment and the ESI-MS analysis were very different, the results revealed that all three zinc-free proteins had bound an alternative metal. Unfortunately, the other two zinc-free proteins (HVO_2901, HVO_A0511) could not be analyzed using ESI-MS because their production levels were too low for this method.

For the four analyzed proteins, the masses of the two peaks were analyzed. Their differences were calculated and the difference between mass peak 2 and the calculated theoretical mass of the metal-free apo-proteins was included. The values are summarized in Table 2. For the positive control HVO_0758, the difference between the two mass peaks was 63.0 Da, and between the metal-containing ESI-MS peak and the calculated mass, the difference was 63.5 Da. As it is known that this protein binds zinc, this value had to be compared with the zinc mass of 64 Da (the most common isotope of zinc), and the values are indeed very similar.

The mass differences for the two proteins HVO_1670A and HVO_A0254A were 59.9 Da (59.7 Da) and 59.9 Da (59.5 Da), respectively. These differences are indicative of the binding of either cobalt (58.9 Da) or nickel (58.5 Da) (see Section 3). The mass difference for the protein HVO_0489 was 68.9 Da (306.9 Da). The difference between the two mass peaks showed that HVO_0489 is also a metal-containing protein, and the large difference between the measured and the calculated mass indicates that HVO_0489 contains, in addition, a post-translational modification (see Section 3).

In summary, three of the five zinc-free C(P)XCG µ-proteins could be analyzed via ESI-MS, and all three turned out to bind an alternative metal instead of being metal-free.

### 2.5. Attempts to Identify the Bound Non-Zinc Metal

It has been reported that the dye zincon is able to bind different metals and that the spectra of the metal–zincon complexes allow the differentiation between bound zinc, cobalt, and copper [41]. To make use of this method, the three isolated zinc-free proteins and the positive control HVO_0758 were treated as described above, to liberate the bound metal, and were incubated with zincon. Unfortunately, the sensitivity of the assay was not high enough for the available protein concentrations and did not allow for the identification of the bound metals.

As an alternative approach, it was attempted to complex the metal with EDTA and to determine the mass of the metal–EDTA complex via mass spectrometry. Unfortunately, this novel and unprecedented approach was also not successful.

In a third attempt to identify the complexed metal, the isolated proteins were treated with EDTA to remove the metal and were dialyzed to remove the metal–EDTA complex. Subsequently, aliquots of the samples were incubated with different metals and were then analyzed via ESI-MS again to reveal which metal could re-establish the original spectrum. Unfortunately, the results were inconclusive.

### 2.6. AlphaFold Structure Predictions and Alignments to NMR Solution Structures

**Figure 4:**
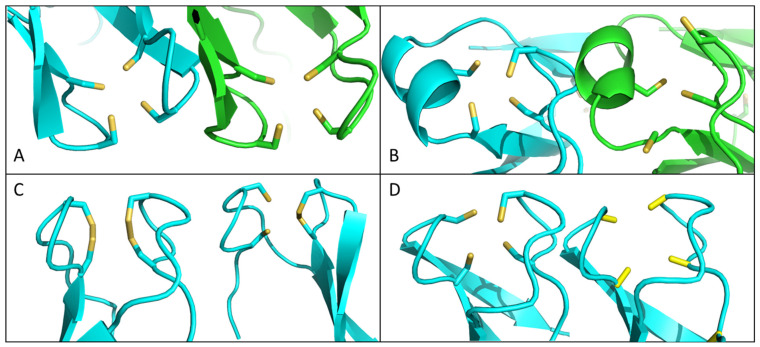
(**A**) Comparison between zinc-binding pocket 2 (ZBP2) of HVO_2753, AlphaFold structure prediction (blue), and the NMR solution structure (green) (PDB: 6YDH). (**B**) Structure comparison of ZBP1 of HVO_2753 between the AlphaFold structure prediction (blue) and the NMR structure (green) (PDB: 6YDH). (**C**) AlphaFold structure predictions of HVO_0546 (left) and HVO_B0212 (right), zoomed into the putative ZBP formed by the C(P)XCG-related motifs. (**D**) AlphaFold structure predictions of the putative zinc-binding pockets of HVO_A0511 (left) and a version of HVO_A0511 in which all four cysteines had been replaced by alanines (right).

AlphaFold2 was introduced only four years ago [42]. It has turned out to be very reliable in the prediction of protein structures, and one might develop the impression that experimental structure elucidation methods have become obsolete. As we have determined the NMR solution structures of HVO_0758 and HVO_2753, it seemed interesting to compare the real structures with the structures predicted using AlphaFold2. For HVO_0758, the two structures were virtually identical. The alpha helix, the beta sheets, and the two hairpins that make up the zinc-binding pocket were correctly predicted. Also, the structure of zinc-binding pocket 2 of HVO_2753, which binds zinc, was faithfully predicted using AlphaFold2 (Figure 4A). In contrast, the real structure and the predicted structure of the zinc-binding pocket 1, which does not bind zinc, differed considerably (Figure 4B). AlphaFold2 predicted that the four cysteines point towards each other and have zinc-binding capacity, while the real structure shows that two of the cysteines point in outward directions and neatly explains why no zinc can be bound by this predicted “zinc finger”. Evidently, the training set of AlphaFold2 (the whole PDB database) did not contain any, or not enough, structures of zinc-free zinc fingers to allow for the prediction of the correct structure. Via Foldseek [43], we tried to find structural homologues of HVO_0758 and HVO_2753. Unsurprisingly, the search engine yielded no results.

This mixed result of the accuracy of AlphaFold2 predictions prompted us to predict the structures of the 12 additional C(P)XCG µ-proteins for which the zinc content could be quantified. In all but two cases, a zinc-binding pocket was predicted with the four cysteines pointing toward each other in a tetrahedral configuration that is indicative of metal binding. However, for HVO_B0212, a disulfide bridge between two of the cysteines was predicted, and for HVO_0546, two disulfide bridges between two pairs of cysteines were predicted (Figure 4C). Thus, in these predictions, not all four cysteines are available to complex a zinc ion. However, both proteins have been experimentally proven to bind zinc (Figure 2), and, thus, these predictions cannot be true.

It seemed interesting to analyze whether or not the AlphaFold2 predictions of a zinc-binding pocket are based on the presence of four cysteines. To this end, a structure prediction of HVO_A0511, including the cysteines, was compared to a prediction of a variant in which the four cysteines had been replaced by alanines. Figure 4D shows that AlphaFold2 predicts a tetrahedral configuration of the four amino acids regardless of their capacity to complex a metal ion. The same result was obtained for two additional examples, HVO_ 0758 and HVO_2400. Therefore, it seems that AlphaFold2’s predictions of zinc fingers do not depend on the presence of possible metal-complexing amino acids (cysteines or histidines) but rest on the overall primary structure similarity with known zinc finger domains.

Although the last “critical assessment of structure prediction” (CASP) conducted in 2022 revealed that AlphaFold2 was superior to all other methods for the prediction of single-protein structures [44], RoseTTAfold2 was also applied as an alternative [45]. The results are shown in Supplementary Figure S2. Like AlphaFold2, RoseTTAfold2 also predicted the tetrahedral conformation of the four cysteines in zinc-binding pocket 1 of HVO_2753, in contrast to the real structure (Supplementary Figure S2B). However, in contrast to AlphaFold2, RoseTTAfold2 did not predict the presence of disulfide bridges for two zinc-containing zinc fingers (Supplementary Figure S2C). Again, similar to AlphaFold2, RoseTTAfold2 predicted a tetrahedral conformation of amino acids even when the four cysteines had been replaced with alanines (Supplementary Figure S2D). The CASP conduct had shown that AlphaFold2 produced the results with the highest quality for two-thirds of all targets. However, for the prediction of zinc fingers, RoseTTAfold2 turned out to be superior at least for one aspect (prediction of thiols versus disulfide bridges).

After the completion of the current project, a new version of AlphaFold was introduced, AlphaFold3 [46]. It was used for the same challenges as the other two programs, and the results are shown in Supplementary Figure S3. Also, AlphaFold3 wrongly predicted a tetrahedral conformation of the four cysteines for the zinc-free “zinc finger” of HVO_2753 (Supplementary Figure S3B). However, in contrast to AlphaFold2, AlphaFold3 did not predict disulfide bridges for the two zinc-containing zinc fingers (Supplementary Figure S3C). Like the other two programs, the identity of the amino acid did not influence the structural prediction of a tetrahedral conformation (Supplementary Figure S3D). Taken together, all three programs, including the newest program from 2024, cannot correctly predict whether the structure formed by two C(P)XCG motifs contains zinc or is metal-free.

## 3. Discussion

Even if it has been recognized in recent years that µ-proteins are ubiquitously present in all species and fulfill important functions, they are still severely understudied compared with average-sized proteins [7,8,13]. This is even more true for C(P)XCG-containing µ-proteins, for which only two examples had been characterized prior to our project, i.e., the transcriptional regulator *brz* from the haloarchaeon *H. salinarum* [16] and rubredoxin from the bacterium *Clostridium pasteurianum* [47,48]. The latter was shown to contain iron instead of zinc, in contrast to the belief that two C(P)XCG motifs are predictive of the presence of a zinc finger (see below).

The project on predicted zinc finger µ-proteins from *H. volcanii* is based on the early observation of the Oesterhelt group that 20 of 380 small proteins from the haloarchaeon *H. salinarum* contain two C(P)XCG motifs [15]. In *H. volcanii*, the number turned out to be even higher, with 49 C(P)XCG proteins in the size range of up to 70 aa. In bacteria, these one-domain predicted zinc finger proteins also exist but at lower ratios than in haloarchaea [16].

As very few similar proteins exist, the bioinformatic predictions of putative functions are not possible. The report about the generation and phenotypic characterization of the deletion mutants of 16 genes for C(P)XCG µ-proteins multiplied the number of predicted zinc finger µ-proteins with known biological roles [29]. Since then, about 20 more deletion mutants have been generated and characterized; manuscript in preparation). The majority of these proteins turned out to be involved in (the regulation of) growth in specific media, biofilm formation, or swarming. Evidently, the proteins are very important for *H. volcanii*, because in many cases, the lack of a single protein of less than 70 aa out of a proteome of more than 4200 proteins led to a loss of function or a gain of function phenotype. The phenotypes also revealed that the proteins are not redundant, and the differences in phenotypes show that the C(P)XCG proteins fulfill different functions in the cell. These results were not unexpected because many (average-sized) zinc finger proteins from eukaryotes have been well characterized and were shown to be involved in many different processes [49,50,51,52,53,54].

High-resolution NMR solution structures have been solved for the proteins HVO_0758 and HVO_2753 [30,31]. Remarkably, it was revealed that only one of the two predicted zinc fingers from HVO_2753 really bound a zinc ion, while the other one did not. This confirmed the result of a prior biochemical zinc quantification with a fluorimetric assay, and the structure neatly explained why this predicted zinc-binding pocket was metal-free, i.e., the four cysteines were not in the tetrahedral conformation required for complex formation but two cysteines pointed in outward directions [31]. The high-resolution NMR solution structures of HVO_2753 and HVO_0758 as well as biochemical assays revealed that one of three predicted “zinc-binding pockets” was in fact metal-free. The binding of zinc to all three sites was not only predicted based on the presence of the C(P)XCG motifs, but AlphaFold2 also did not correctly predict the metal-free site, albeit the overall structures of both proteins were predicted rather well (RMSD of 2.1 Å). In addition, another program, RoseTTAfold2, and the newest version of AlphaFold, AlphaFold3, also could not correctly predict the structure of the metal-free site. Therefore, in spite of the immense power of current AI-based structure prediction programs, the experimental quantification of zinc binding still remains necessary to unravel whether or not a predicted zinc finger is occupied or metal-free.

Therefore, the aim of this project was to determine the zinc content of as many C(P)XCG µ-proteins of *H. volcanii* as possible.

This required the production and purification of many different proteins. We aimed to produce the proteins homologously in *H. volcanii* to ensure the proper folding of the proteins in the high-salt cytoplasm. For the two proteins that had been purified previously, HVO_0758 and HVO_2753, it had been shown that they also fold in the low-salt cytoplasm of *E. coli* and that the structures are identical after heterologous and homologous production. However, this need not be true for all C(P)XCG µ-proteins. In addition, the proteins should be produced in the presence of the native divalent metal concentrations in *H. volcanii*. This could also be important because it has been shown that the rubredoxin from *C. pasteurianum* not only binds iron in its native host but also zinc after heterologous production in *E. coli* [55].

For the homologous production, 45 genes were cloned in two versions encoding a protein with an N-terminal or a C-terminal His_6_-tag, respectively, so that 90 expression plasmids were generated. The shuttle vector pSD1-R1/6 was used as a backbone, which contains a strong constitutive promoter [32]. Unfortunately, it turned out that only 14 of the 45 proteins could be produced in levels high enough for purification and zinc quantification. The N-terminal His_6_-tag had a higher success rate than the C-terminal His_6_-tag, and only very few proteins could be produced with both tags (Supplementary Table S1). The inability to produce the majority of the 45 proteins is a specific problem in the production of µ-proteins because the vector pSD1-R1/6 had been previously used in other projects with normal-sized proteins, and a high production rate and protein yield after purification was successful in all cases. Examples of this are the elucidation of the protein–protein interaction networks of translation initiation [56] and of gene conversion (Sandner and Soppa, in preparation), which, together, required the production and purification of about 50 proteins.

Not only has the production of µ-proteins from haloarchaea often turned out to be problematic but similar drawbacks have also been experienced with many other µ-proteins from bacteria and eukaryotes [1,4,9,15]. For example, in the Priority Program SPP 2002, it was attempted to produce and purify 27 µ-proteins from two archaeal and seven bacterial species. Eight proteins could not be produced at all, while the remaining proteins could be produced to some extent. Only three of the proteins were fully structured (all from *H. volcanii*), while the proteins from the remaining eight species were fully or partially unstructured (molten globule) after heterologous production in *E. coli* [57]. The inability to produce µ-proteins heterologously or homologously can have different explanations. For example, they might be intrinsically unstable because the native concentrations of many µ-proteins are rather low. Or, they might be disordered in the absence of a binding partner, and the binding partner might be totally missing because the respective gene is not expressed under the production conditions or it is not present in the heterologous host. Or, the fusion tag might influence the correct folding of the protein, and the misfolded protein is rapidly degraded. The His_6_-tag is very short, but for µ-proteins, it makes up a non-negligible fraction of the fusion protein (more than 10% for proteins smaller than 60 aa). Or, the mRNA might be very unstable because it contains RNase recognition motifs for the heterologous host or because the 5′-UTR and 3′-UTR are missing. Evidently, there are many factors that can lead to the inability to produce a protein, and this is especially true for µ-proteins.

Several attempts were made to enable the production of proteins that could not be produced using the chassis pSD1-R1/6. As an alternative expression vector, the plasmid pTA929 was used, which contains an inducible promoter [33], but this was not successful. In addition, selected genes were fused with the gene for the normal-sized protein DHFR, which can be easily produced in large quantities in *H. volcanii*. A motif for the TEV protease was inserted between the DHFR and the µ-protein to enable the liberation of the latter. However, for unexplained reasons, the fusion protein formed insoluble precipitates, making isolation impossible. Omitting the usage of an expression plasmid was also tried. Instead, the fusion gene was integrated into the chromosome via homologous recombination. This also did not result in the production of the respective protein. Heterologous production in *E. coli* was also tried, even if the binding of the native metal could not be guaranteed. However, only one of eight proteins could be produced, and this protein could also successfully be produced in *H. volcanii* [58,59]. In the end, attempts for alternative production approaches were terminated, and the project was limited to the 14 proteins that could be produced and purified.

A very sensitive zinc assay exists that makes use of the fluorophore ZnAF-2F [37]. It is highly specific for zinc, and even a more than 1000-fold excess of other divalent metals does not compromise the results. It had already successfully been used to quantify the zinc content of HVO_0758 and HVO_2753 [30,31], and it was applied for the characterization of the 12 isolated proteins in the current project. It turned out that only nine of the proteins complexed a zinc ion, while five proteins were zinc-free. The inability to determine zinc might have several different explanations, even when the native protein is a zinc binder. For example, the affinity between zinc and the apo-protein might be very low (even if that would be unusual for a zinc finger protein). The bound zinc ion might become lost during the two-step isolation procedure, which includes extensive washing steps during affinity isolation and a high dilution during gel filtration. Alternatively, the fusion of the affinity tag might induce a non-native folding, which results in a loss of the high affinity of the protein to zinc. Other explanations are (1) the protein is natively metal-free and (2) a different divalent metal than zinc is bound. To differentiate between these two possibilities, ESI-MS was applied.

ESI-MS is a very gentle method (in contrast to MALDI-MS) that has the ability to preserve metal–protein complexes and determine the native mass of the complex. It has been repeatedly used for the characterization of zinc finger proteins (35 papers with “zinc finger” and “ESI-MS” in title/abstract in PubMed). One disadvantage of the characterization of haloarchaeal proteins is that the method is sensitive to even very low concentrations of NaCl of 1 mM or below [60,61]. These impurities lead to the broadening of the signals and a large decrease in signal intensities. The native isolation of the haloarchaeal proteins was performed in the presence of 2100 mM NaCl, and it is clear that even extensive ultrafiltration against a buffer lacking NaCl could not reduce the concentration to absolutely zero. As discussed above, even dialysis against a buffer lacking NaCl might lead to the loss of a bound metal. In addition, it is not known how haloarchaeal proteins behave in a vacuum. Therefore, aliquots of the samples were treated with EDTA prior to the measurements, and only differences in the spectra with and without this treatment were taken as proof of metal binding. The protein demand is much higher for ESI-MS than for the highly sensitive fluorimetric zinc assay; therefore, the analysis could only be performed for three of the five zinc-free proteins. All three proteins turned out to bind another metal than zinc, and none of the proteins was metal-free. For the positive control HVO_0758, the mass difference between the experimentally determined mass for the metal complex and the calculated mass of the apo-protein was 63.5 Da (Table 2). The mass of the major zinc isotope (48.6%) is 64 Da and, thus, the congruence between the two values is very good.

The differences between the measured values for the metal-containing proteins and the calculated values for the metal-free proteins were close to 60 Da for HVO_1670A and A0254A. These values are rather close to the masses of the major isotopes of cobalt (59 Da, 100%) and nickel (58 Da, 68%). Other possible metals are iron (56 Da, 91%) and copper (63 Da, 69%). Calcium (40 Da, 97%) and cadmium (114 Da, 29%) can be excluded because their molecular masses are too different. In addition, calcium can also be excluded because it requires a different coordination space (6–7 ligands, oxygen ligands) and could not be accommodated in the tetrahedral conformation offered by four cysteines. An additional reason to exclude cadmium is the simple fact that it was not added to the medium. In contrast, all possible metals were available to *H. volcanii* because a trace element solution had been added to the medium to exclude that artificial metal-free sites could be generated due to the lack of the cognate metal ion. Unfortunately, several attempts to identify the bound metal experimentally failed.

The case of protein HVO_0489 is more difficult. The mass difference between the EDTA-treated and untreated samples was 68.9 Da, and this is far from the mass of any metal that could possibly be bound. However, the large difference in the experimentally determined mass and the theoretically calculated mass revealed that the protein does not only complex a metal but is also post-translationally modified. As an alternative approach to identifying the bound metal, putative post-translational modifications (PTMs) were taken into account. It has been shown that post-translational phosphorylations are not uncommon for haloarchaeal proteins [62,63,64]. Therefore, as one possibility, it could be assumed that HVO_0489 carries three phosphorylations at its serine residues. If the mass of three phosphate groups were subtracted from the measured value of mass peak 2, the mass difference between the measured metal-containing protein and the calculated mass of the metal-free apo-protein would be 58.9 Da. This would also be best compatible with a bound cobalt (59 Da) or nickel (58 Da) ion.

Several attempts have been made to identify the bound non-zinc metals of the three proteins HVO_0489, HVO_1670A, and HVO_A0254A. Unfortunately, none of these attempts was successful. Therefore, future studies will be needed to obtain experimental proof of the identity of the complexed metal. However, notably, we could clearly show that these three C(P)XCG µ-proteins are not metal-free.

Various studies exist showing that metals like cobalt, copper, nickel, or cadmium can bind to zinc finger domains of bacterial or eukaryotic proteins [65,66,67,68,69,70]. However, in these cases, the metal-free apo-proteins were incubated with different metals, binding was analyzed, and these metals were not complexed in the native proteins in vivo. A literature search did not reveal a single example that revealed that another metal than zinc was natively binding to a eukaryotic C(P)XCG protein in vivo. In contrast, the mutagenicity and toxicity of copper or cadmium was repeatedly attributed to the fact that it can replace the native zinc in zinc finger proteins, leading to their inactivation [71,72,73,74].

To our knowledge, the only C(P)XCG protein that has been shown to complex another metal is rubredoxin of the bacterium *C. pasteurianum*, which has been proposed to bind iron [48]. However, it is unlikely that the three non-zinc C(P)XCG proteins of *H. volcanii* bind iron (molecular mass of 56 Da). Therefore, the results presented above indicate that they represent the first zinc finger proteins that do not bind zinc or iron.

Notably, there are several examples of predicted zinc finger proteins that have been shown to be natively metal-free [75], like one of the zinc-binding domains of HVO_2753. There are several proteins of the bacterial Ros family that do not bind zinc but have retained their ability to bind DNA and act as transcription factors [75]. It has been proposed that in evolution, additional interactions had stabilized the overall fold of these proteins so that mutations that resulted in the loss of the zinc-binding capacity did not abolish the stability of the protein structure or the function. It will be interesting to reveal in the future how many of the predicted zinc finger proteins are in reality metal-free because they do not need the essential stabilizing role of a central zinc ion anymore. As stated above already, experimental clarification is required because predictions via AlphaFold2, RoseTTAfold2, or AlphaFold3 are not always reliable. Predictions of the overall folding are quite accurate; however, the prediction of the tetrahedral orientation of the four cysteines yielded both false positive and false negative results.

Therefore, an optimization of the method for the homologous production of the C(P)XCG µ-proteins of *H. volcanii* will be necessary. Possibilities include the usage of alternative tags, the addition of long 5′- and 3′-UTRs to stabilize the mRNA, the testing of various other proteins than DHFR as fusion partners, the mutation of the N-terminal and the C-terminal amino acid to stabilize the protein, and more.

Table 3 gives an overview of the results that were obtained in this and two previous studies. Among the 14 proteins that could be produced homologously at all, there was a strong correlation between the production level and the zinc content. All nine proteins that contained zinc had a medium or high production level and, the other way around, all proteins with medium or high production levels contained zinc. All three proteins that contained an alternative metal had low production levels, and for two zinc-free proteins, the production level was so low that it could not be decided via ESI-MS whether they were metal-free or also bind an alternative metal.

Importantly, the characterization of the first 14 predicted zinc finger µ-proteins already revealed that only 9 of them actually did bind zinc, while a non-negligible fraction of 5 proteins did not. From these, three proteins were shown to bind another divalent metal ion, most likely cobalt or nickel. In addition, one of two zinc-binding motifs of HVO_2753 was shown to be metal-free. Therefore, the variability in bioinformatically predicted zinc finger domains seems to be higher than anticipated. Thus, it seems worthwhile to analyze various additional predicted zinc finger proteins from other archaea, bacteria, and eukaryotes to unravel whether this variability is confined to haloarchaea or whether it is more widespread and has been overlooked until now. This is important because predicted zinc finger proteins are involved in many essential functions, especially in eukaryotes including humans, and in this study, we could clearly show that predicted zinc fingers can be zinc-free or complex other metals than zinc.

## 4. Materials and Methods

### 4.1. Databases and Bioinformatics Analyses

All gene and protein sequences from the *H. volcanii* genome were retrieved from the HaloLex database [76]. The cloning experiments were planned with CloneManager (version 8.0).

The AlphaFold2 structure predictions of 13 of the studied zinc finger µ-proteins were downloaded from their respective pages of the AlphaFold website (alphafold.com (accessed on 1 May 2024)) [42]. There was no structure prediction for HVO_1670A available. Therefore, a prediction was generated using the ColabFold tool via ChimeraX [77,78]. AlphaFold3 structure predictions were generated via the AlphaFold3 webserver tool (alphafoldserver.com (accessed on 1 May 2024)) [46]. RoseTTAFold2 structure predictions were generated via the RoseTTAFold2 notebook in ColabFold [45] (https://doi.org/10.1101/2023.05.24.542179 (accessed on 1 May 2024)).

Foldseek was used to search for homologous structures [43]. Structure alignments were performed via PyMOL (PyMOL Molecular Graphics System, Version 2.0, Schrödinger, LLC, New York, NY, USA).

### 4.2. Strains, Media, and Culture Conditions

The *E. coli* strain XL1-Blue MRF’ (Agilent Technologies, Waldbronn, Germany) was used for cloning. Standard molecular genetic techniques were used [79].

All *H. volcanii* strains generated in this study were derived from the strain H26 [80]. The strains were grown in a complex medium with 0.5 µg/mL of novobiocin [32,81]. A trace element solution was added to ensure that all metals that could possibly bind to two C(P)XCG motifs were available to *H. volcanii* and precluded such motifs from remaining artificially metal-free due to a lack of the cognate metal. In short, the medium contained 2.14 M of NaCl, 135 mM of KCl, 220 mM of MgCl_2_, 40 mM of MgSO_4_, 9 mM of CaCl_2_, 0.3% (*w*/*v*) yeast extract, 0.5% (*w*/*v*) tryptone, 50 mM of Tris/HCl pH7.2, 3.5 µM of ZnSO_4_, 1.5 µM of MnCl_2_, 50 µM of H_3_BO_3_, 8.5 µM of CoCl_2_, 0.5 µM of CuCl_2_, 1 µM of NiCl_2_, 1 µM of Na_2_MoO_4_, and 8 µM of FeSO_4_.

### 4.3. Homologous Production and Native Purification

All production strains were generated by cloning the respective genes together with codons for either an N- or C-terminal His_6_-tag into the shuttle vector pSD1-R1/6, which contains a strong constitutive promoter [32]. For some genes, a modified version was used that contained an NdeI site instead of the NcoI site of the original plasmid [31]. All primers used for the amplification and cloning of the genes are listed in Supplementary Table S2. The faithful cloning was verified by sequencing (custom GATC sequencing service from Eurofins).

The vectors were then used to transform either the corresponding deletion mutant or, if unavailable, the wildtype H26. The integrity of the plasmid in the resulting production strains was rechecked by amplifying the cloned region with vector-specific primers and sequencing.

For protein production, the first pre-cultures of the respective strains were grown overnight in complex media with 0.5 µg/mL of novobiocin. A total of 0.5 to 1 L of complex medium with 0.5 µg/mL pf novobiocin were then inoculated with a start OD_600_ of 0.005. The production cultures were grown for at least 24 h to an OD_600_ of more than 1.5. The cells were harvested via centrifugation (6500 g for 30 min at 4 °C) and the supernatant was removed. The cells were resuspended in binding buffer (2.1 M of NaCl, 20 mM of Hepes, pH 7.5, 20 mM of imidazole, 1 mM of PMSF) and lysed via sonication. The proteins were purified via a two-step purification process as described previously [30]. The first step was affinity chromatography with nickel chelating sepharose, the second step was size exclusion chromatography using a SuperDex 75 FPLC column. The purity was checked using Tricine gels [82].

### 4.4. Fluorimetric Zinc Quantification

The quantification of bound zinc was performed using the highly zinc-specific and very sensitive fluorophore ZnAF-2F [37]. The proteins were purified as described above and protein concentration was determined via UV absorption at 280 nm. The experiments were performed as described previously [31]. In short, 1 μM of the protein was dialyzed against 25 mM of NaCl and 20 mM of HEPES, pH 7.5, to reduce the salt concentration. Then, it was incubated with proteinase K (100 μg/mL) overnight at 37 °C to hydrolyze the protein and release the bound zinc. Then, 4 μM of the fluorophore ZnAF-2F was added and the fluorescence was quantified using a microtiter plate fluorimeter (ClarioStar, BMG LabTech, Ortenberg, Germany). An excitation wavelength of 492 nm and a detection wavelength of 517 nm were used. Four technical replicates were used for each biological replicate. A standard curve was generated that consisted of zinc concentrations from 0 µM to 2 µM of ZnCl_2_. When the zinc concentration of a protein was close to zero, the measurement was repeated with internal standardization. To this end, 1 µM of ZnCl_2_ was added and it was verified that the added zinc concentration could faithfully be quantified. At least three biological replicates were performed for each protein.

### 4.5. Electrospray Ionization Mass Spectroscopy

All proteins used in ESI-MS experiments were produced and purified as described above. Protein solutions with concentrations of 10–50 µM were buffer exchanged into 100 mM of ammonium acetate (pH 7.2) using Amicon Ultra 0.5 Centrifugal Filters with 3 kDa MWCO (Merck KGaA, Darmstadt, Germany). Half of the sample was treated with 100 µM of EDTA for 30 min in order to remove the possibly bound metal ions. Measurements with and without EDTA treatment were used to determine if there were mass differences in the protein due to the loss of a metal ion. The ESI-MS measurements were performed using a Synapt G2-S instrument (Waters Corp., Manchester, UK) equipped with a 32 kDa quadrupole, operating in positive nESI mode. Specific instrument voltages and configurations are detailed below. Capillary voltages ranging from 1.8 to 2.2 kV were used, along with a sample cone voltage and source offset set between 100 V and 150 V. Collision voltages within the trap cell were adjusted between 10 V and 50 V. Changes in the collision energy showed no significant impact on metal binding and were, therefore, used only for reductions in salt attachments. nESI tips were made in-house from borosilicate glass capillaries using a Flaming/Brown Micropipette Puller (P-1000; Sutter Instrument Co., Novato, CA, USA) and subsequently coated with gold using a sputter coater (Q 150R S, Leica Microsystems, Wetzlar, Germany).

## 5. Conclusions

The genome of *H. volcanii* encodes 49 µ-proteins with two C(P)XCG motifs that are indicative of the formation of zinc fingers. In total, 14 of these proteins could be produced homologously as His_6_-tagged versions in amounts that were high enough for biochemical analyses. The success or failure of production could not be rationalized based on size, pI, or C(P)XCG motifs. The quantification of the zinc content of the purified proteins revealed that a majority of nine proteins indeed complexed one equivalent of zinc, as predicted. In contrast, five proteins turned out to be zinc-free. Three of the latter proteins could be analyzed via ESI-MS, and all of them turned out to complex an alternative metal ion. The mass differences between metal-containing and EDTA-treated samples indicated that these proteins most probably complex nickel or cobalt, but definite proof for this is still missing. Experimental approaches to determining the metal content of predicted zinc finger proteins are necessary because even up-to-date AI-driven prediction programs like AlphaFold2, RoseTTAfold2, or AlphaFold3 cannot always correctly predict whether a structure is metal-containing or in reality metal-free.

## Figures and Tables

**Table 1:** Length distribution of all proteins and C(P)XCG proteins in the genome annotation of *H. volcanii*.

Length (aa)	No. Total	No. with C(P)XCG Motifs	% C(P)XCG Motifs
<30	18	0	0
30–40	54	5	9.3
40–50	84	15	17.9
50–60	108	20	18.5
60–70	133	9	6.8
70–80	105	11	10.5
80–90	104	5	4.8
90–100	120	9	7.5
>100	3496	20	0.6
**total**	**4222**	**94**	**2.2**

**Table 2:** Calculated masses obtained for the metal-free (peak 1) and metal-bound species (peak 2) of the µ-proteins via ESI-MS.

Protein and Tag	Theoretical Mass [Da]	Mass Peak 1 [Da]	Mass Peak 2 [Da]	Δ Mass [Da] *^1^
HVO_0758_CHis	7459.4	7455.9	7522.9	63.0 (63.5)
HVO_0489_NHis	6130.7	6364.7	6437.6	68.9 (306.9)
HVO_1670A_NHis	5640.2	5636.0	5699.9	59.9 (59.7)
HVO_A0254A_NHis	6161.6	6157.2	6221.1	59.9 (59.5)

*^1^ The numbers in brackets are the difference between the measured mass peak 2 (metal-containing protein) and the calculated theoretical mass of the metal-free protein. For the calculation of the mass differences of the measured masses of the metal-containing and the metal-free proteins, it was taken into account that in the metal-free apo-protein the four cysteines are probably oxidized to two disulfide bonds and the protein is, thus, missing four protons (4 Da) that are present in the metal-containing reduced protein.

**Table 3:** Summary of all putative zinc finger µ-proteins that have been used in the fluorimetric zinc-binding assay. The tag used for production and purification, the Uniprot accession number, the C(P)XCG motifs, the measured amount of zinc, and the results of the ESI experiments are given. Zinc assay data from HVO_0758 and HVO_2753 are taken from [30] and [31], respectively. N.a.—not analyzed.

Protein Name and Tag	Accession No.	Yield *^2^	Amount of Zinc Bound	Other Metal Bound?
HVO_0098 (N-His)	D4GYU3	medium	1	-
HVO_0489 (N-His)	D4GS29	low	0	yes
HVO_0546 (N-His)	D4GSE3	medium	1	-
HVO_0758 (N-His)	D4GTQ1	high	1	-
HVO_0767 (N-His)	D4GTR5	medium	1	-
HVO_1352 (N-His)	D4GXN5	high	1	-
HVO_1670A (N-His)	A0A8E8PIY1	low	0	yes
HVO_2400 (C-His)	D4GWP7	medium	1	-
HVO_2753 (N-His) (ZBP1)	D4GWB3	high	0	no metal
HVO_2753 (N-His) (ZBP2)			1	-
HVO_2901 (N-His)	D4GXP6	low	0	n.a.
HVO_A0254A (N-His)	D4GQT4	low	0	yes
HVO_A0511 (N-His)	D4GRH3	low	0	n.a.
HVO_B0212 (N-His)	D4GPL3	high	1	-
HVO_C0086 (C-His)	D4H0G6	medium	1	-

*^2^ Yield per liter of culture: low: <0.1 mg; medium: 0.1–1 mg; high: 1–4 mg.

## Data Availability

All results are part of this manuscript or the Supplementary Materials. Strains and plasmids are freely available upon request.

## Supplementary Materials

The following supporting information can be downloaded at https://www.mdpi.com/article/10.3390/ijms25137166/s1.
