# Supplementary material for: C(P)XCG Proteins of Haloferax volcanii with Predicted Zinc Finger Domains: The Majority Bind Zinc, but Several Do Not
Source: Int J Mol Sci. 2024 Jun 28;25(13):7166. doi: 10.3390/ijms25137166 (PMC11241148; doi:10.3390/ijms25137166)
Supplement: Supplementary file 1 [file ijms-25-07166-s001.zip › SI Figures.pdf]

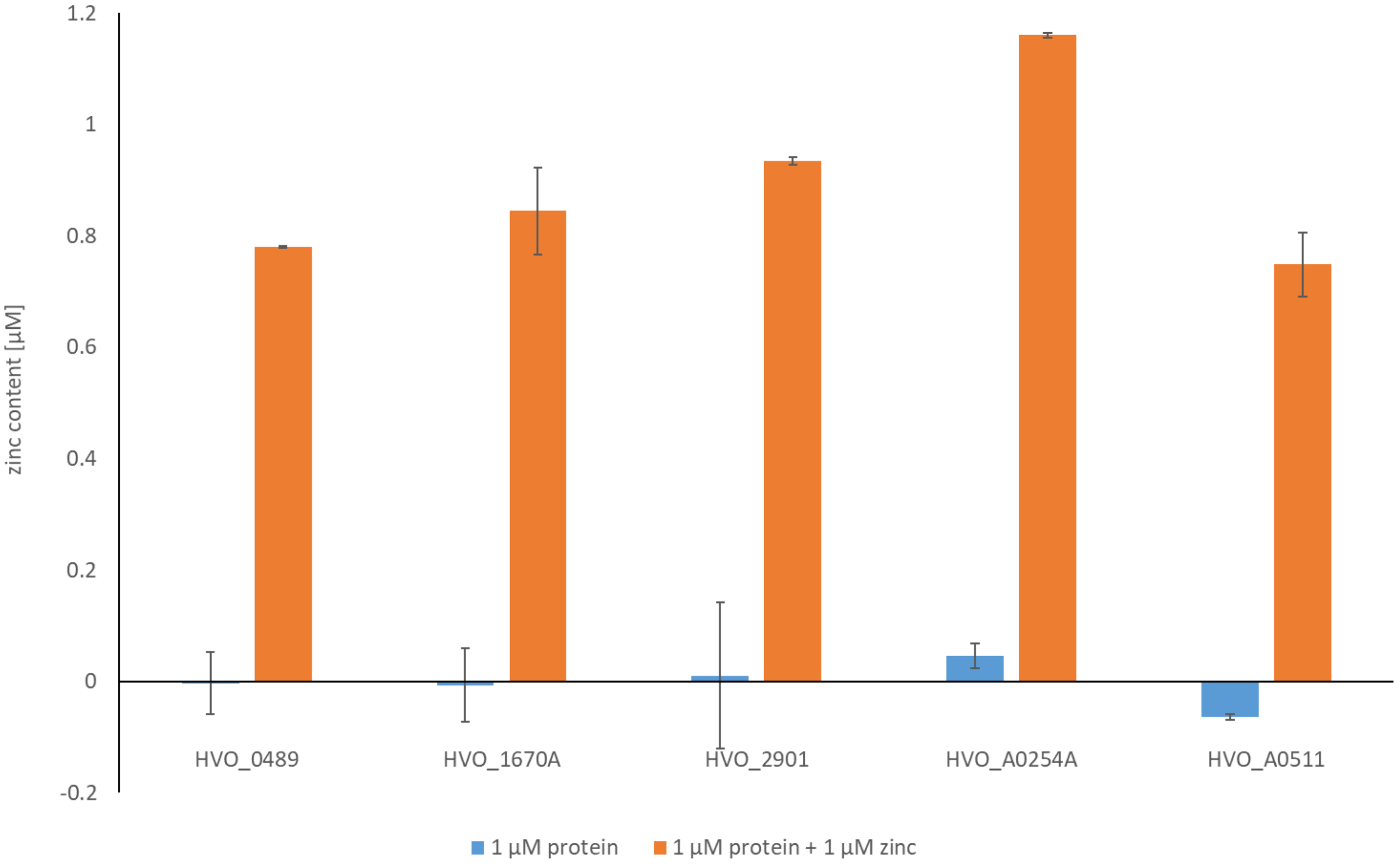

Figure S1: Measured zinc content for 1 µM of protein before (blue) and after (orange) addition of 1 µM Zn^{2+}. Shown are mean values and standard deviations of three biological replicates.

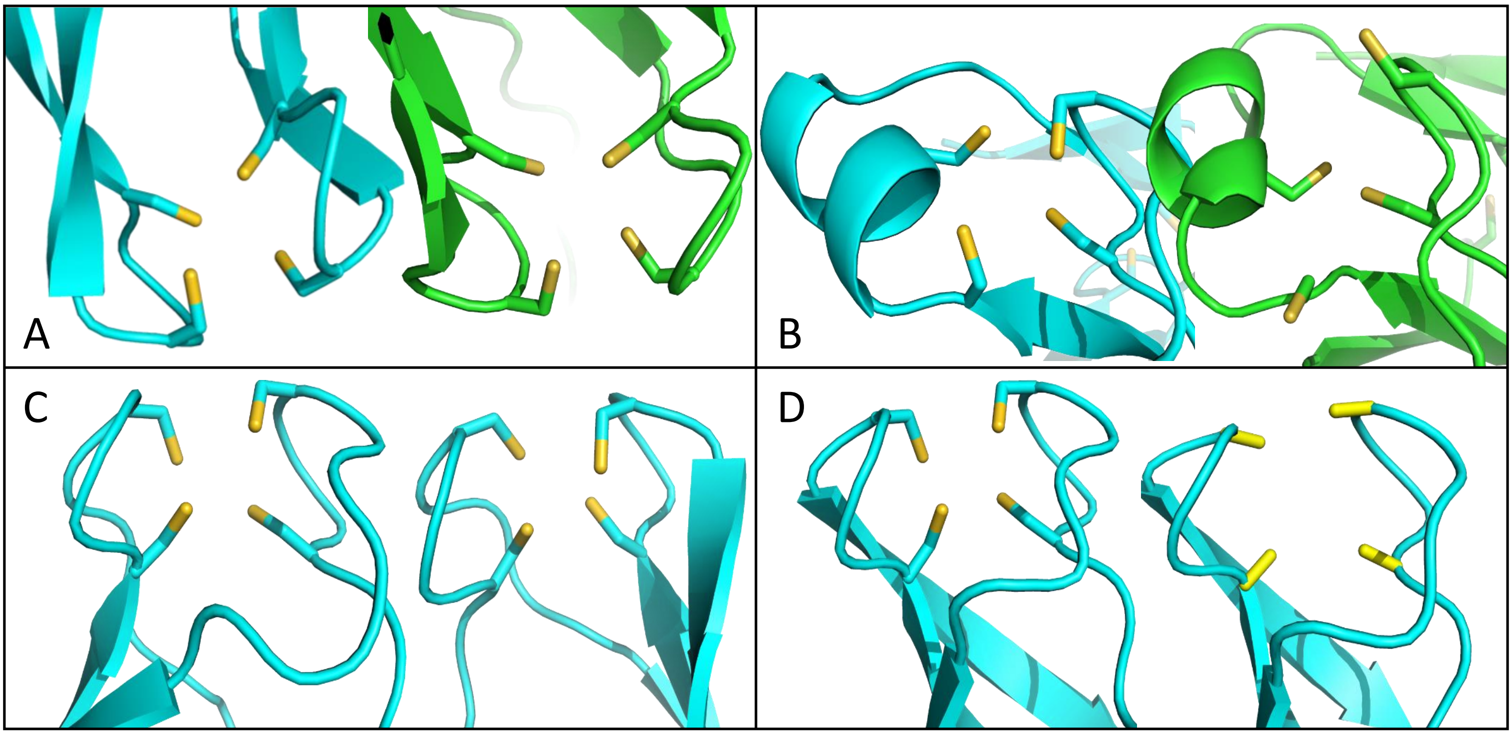

Figure S2: A) Comparison between zinc binding pocket 2 (ZBP2) of HVO_2753, RoseTTAFold2 structure prediction (blue) and the NMR solution structure (green) (PDB: 6YDH). B) Structure comparison of ZBP1 between the RoseTTAFold2 structure prediction (blue) and the NMR structure (green) (PDB: 6YDH). C) RoseTTAFold2 structure predictions of HVO_0546 (left) and HVO_B0212 (right), zoomed into the putative ZBP formed by the C(P)XCG-related motifs. D) RoseTTAFold2 structure predictions of the putative ZBPs of HVO_A0511 (left) and a version of HVO_A0511 in which all four cysteines have been replaced by alanines (right).

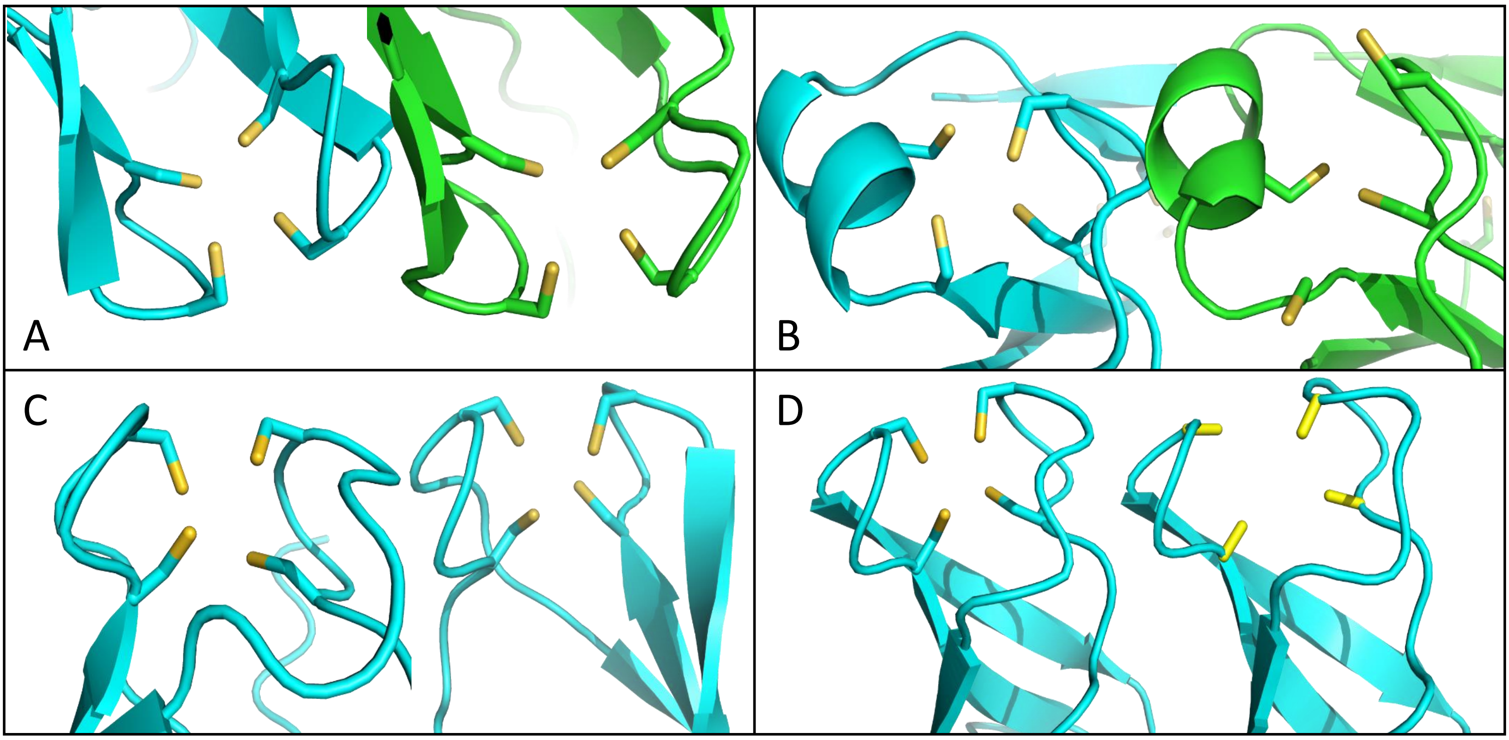

Figure S3: A) Comparison between zinc binding pocket 2 (ZBP2) of HVO_2753, AlphaFold3 structure prediction (blue) and the NMR solution structure (green) (PDB: 6YDH). B) Structure comparison of ZBP1 between the AlphaFold3 structure prediction (blue) and the NMR structure (green) (PDB: 6YDH). C) AlphaFold3 structure predictions of HVO_0546 (left) and HVO_B0212 (right), zoomed into the putative ZBP formed by the C(P)XCG-related motifs. D) AlphaFold3 structure predictions of the putative ZBPs of HVO_A0511 (left) and a version of HVO_A0511 in which all four cysteines have been replaced by alanines (right).
